# Calculating Load and Intensity Using Muscle Oxygen Saturation Data

**DOI:** 10.3390/sports12040113

**Published:** 2024-04-22

**Authors:** Aldo Vasquez-Bonilla, Rodrigo Yáñez-Sepúlveda, Carlos D. Gómez-Carmona, Guillermo Olcina, Jorge Olivares-Arancibia, Daniel Rojas-Valverde

**Affiliations:** 1 Grupo de Avances en Entrenamiento Deportivo y Acondicionamiento Físico, Facultad de Ciencias del Deporte, Universidad de Extremadura, 10003 Caceres, Spain; alvasquezb@unex.es (A.V.-B.); golcina@unex.es (G.O.); 2 Faculty Education and Social Sciences, Universidad Andres Bello, Viña del Mar 2520000, Chile; rodrigo.yanez.s@unab.cl; 3 Grupo de Optimización del Entrenamiento Deportivo, Facultad de Ciencias del Deporte, Universidad de Extremadura, 10003 Caceres, Spain; cdgomezcarmona@gmail.com; 4 Grupo AFySE, Investigación en Actividad Física y Salud Escolar, Escuela de Pedagogía en Educación Física, Facultad de Educación, Universidad de las Américas, Santiago 8320000, Chile; 5 Centro de Investigación y Diagnóstico en Salud y Deporte (CIDISAD), Escuela Ciencias del Movimiento Humano y Calidad de Vida (CIEMHCAVI), Universidad Nacional, Heredia 863000, Costa Rica; drojasv@hotmail.com; 6 Núcleo de Estudios en Alto Rendimiento y Salud (NARS), Escuela del Movimiento Humano y Calidad deVida (CIEMHCAVI), Universidad Nacional, Heredia 863000, Costa Rica

**Keywords:** muscle oxygenation, training load, exercise efficiency, exercise physiology and physical performance

## Abstract

The study aimed to calculate training intensity and load using muscle oxygen saturation (SmO_2_) during two differentiated physical tasks. 29 university athletes participated in a 40-m Maximal Shuttle Run Test (MST, 10 × 40-m with 30 s recovery between sprints) and a 3000-m time trial run. Distance and time were used to calculate external load (EL). Internal load indicators were calculated based on percentage of maximum heart rate (%HR_MAX_) and SmO_2_ variables: muscle oxygen extraction (∇%SmO_2_) and the cardio-muscle oxygen index (CMOI) was also provided by relating ∇%SmO2 ÷ %HR_MAX_, and the training load were calculated as the product of speed (m/min × IL) and the efficiency index [Eff_index_ (m/min ÷ IL)]. A student t test was applied based on Bayesian factor analysis. As expected, EL differed in the 40-m MST (331 ± 22.8) vs. 3000-m trials (222 ± 56.8) [BF^10^ = 6.25e^+6^; *p* = <0.001]. Likewise, IL showed higher values in 40-m MST (39.20 ± 15.44) vs. 3000-m (30.51 ± 8.67) in CMOI: [BF^10^ = 1.70; *p* = 0.039]. Training load was greater in 40-m MST (85.77 ± 27.40) vs. 3000-m (15.55 ± 6.77) [(m/min × ∇%SmO_2_): BF^10^ = 12.5; *p* = 0.003] and 40-m MST (129.27 ± 49.44) vs. 3000-m (70.63 ± 32.98) [(m/min × CMOI): BF^10^ = 169.6; *p* = <0.001]. Also, the Eff_index_ was higher in 40-m MST (10.19 ± 4.17) vs. 3000-m (6.06 ± 2.21) [(m/min × ∇%SmO_2_): BF^10^ = 137.03; *p* = <0.001] and 40-m MST (9.69 ± 4.11) vs. 3000-m (7.55 ± 1.87) [(m/min × CMOI): BF^10^ = 1.86; *p* = 0.035]. This study demonstrates calculations of training intensity and load based on SmO_2_ as an internal load indicator along with speed as an external load indicator during two differentiated exercises.

## 1. Introduction

According to Pillitteri et al. [1], intensity should be considered as a relative measure due to individual athlete responses (i.e., through physiological indicators of internal load) or the training outcome (i.e., external load). The meaning of intensity should not be confused with a load indicator (e.g., speed above 25 km·h^−1^) that can only explain if an activity can be considered “intense” compared to another activity (e.g., speed between 0 and 8 km·h^−1^) [1].

The concept of intensity may be similar to the training load exposed by the athlete which represents the demand of the task [2]. However, exercise scientists define training load specifically as the combination of volume and intensity of training. In this sense, training load refers to the relationship between the quantity of exercise performed and its relative intensity (i.e., the accumulated dose of training exposure) [3]. Therefore, high training loads involve weeks, sessions or activities with high volumes, high intensities, or both. In contrast, low training loads refer to periods of relatively low volume, low intensity, or both [2]. In addition, to consider training load different from intensity, the variation in exercise modality must be taken into account; for example, long duration exercise (e.g., >1 h steady pace) vs. short duration repetition exercise (e.g., sprints, High-Intensity Interval Training or Sprint Interval Training). The imposed load of the exercise modality depends on the metabolism and energy substrate used and the athlete’s ability to utilize that energy substrate [4,5]. In conclusion, intensity is wrapped within the context of training load, which needs both external and internal indicators to be explained during training.

Likewise, an athlete’s physical performance can be evaluated by assessing the relationship between external indicators and internal ones [6], which also known as the training load index or efficiency index (Eff_index_). An athlete’s performance depends on their internal response to the exposed training load [7], as opposed to the intensity of the training load itself, which refers to the product of the external load and physiological indicators (internal load). To monitor external load (EL) time units or speed (distance ÷ time) and power (force × velocity) obtained from a task, session or physical exercise can be considered [8]. Percentages of heart rate maximum (%HR_MAX_), maximal oxygen consumption (%VO_2_max), and rating of perceived exertion (RPE) have usually been used as internal load indicators [8]. However, from a physiological viewpoint, the %HR_MAX_ is the most studied and easy-to-monitor parameter for internal load, but it has limitations in identifying metabolism or energy substrate used during exercise. It also has a high dependence on the autonomic nervous system, which can be imbalanced by emotions, stress, depression, etc. [9].

To overcome this problem of %HR_MAX_, the muscle oxygen saturation (SmO_2_) is a better parameter to measure the internal response of the athletes [10]. SmO_2_ represents the balance between local muscle oxygen supply and the metabolic oxygen demand [11]. SmO_2_ is derived from hemoglobin parameters [((oxyhemoglobin/(oxyhemoglobin + deoxyhemoglobin)) × 100)] and is measured with portable sensors that use non-invasive near-infrared spectroscopy (NIRS) technology [12]. NIRS uses the Lambert-Beer law logarithms and has been extensively studied to measure training adaptations in different sports [13]. During exercise, commonly the SmO_2_ desaturation and resaturation are used as performance indicators, since efficient oxygen transport during exercise is key to maintaining intensity [10]. Also, SmO_2_ provides a dynamic physiological delimitation that identifies between sustainable and unsustainable exercise [14], and is correlated with VO_2_ (L/min) (*r* = 0.943) and energy expenditure (Kcal/min) (*r* = 0.949). For these reasons, SmO_2_ is proposed as a better indicator than HR because when entering in a high-intensity zone its behavior is non-linear [10] obtaining small changes that can be important to analyze [15,16].

Currently, SmO_2_ has not been included in calculating intensity indexes due to SmO_2_ changes during exercise being attributed to vascular factors like neural reflexes occurring in vasoconstriction and vasodilation during exercise, which cannot be controlled practically during exercise [17,18]. An example of this phenomenon is the decreased intramuscular Partial Pressure of Oxygen (PO_2_) provoked by intense exercise [19]. While these factors are individual, they cannot be used as training variables or even measured during daily physical activity. Still, recent studies have further validated the interpretation of SmO_2_ as an indicator of muscle bioenergetics, offering formulas that are accessible to coaches and athletes [10,16,20,21]. One of the biggest challenges for scientists and coaches collecting training data is being able to analyze it to make meaningful inferences. For this reason, the SmO_2_ formulas will be presented in the methodology section of this study.

Finally, to understand the use of SmO_2_ as an indicator of training intensity and load, it is appropriate to consider the time or distance of the exercises (i.e., the volume) to provide data on the training effect [3]. In this sense, the model proposed by Pillitteri et al. [1] can be used to calculate training load intensity by multiplying external load by internal load and to calculate the efficiency index (Eff_index_) by dividing external load by internal load. Therefore, to assess the use of SmO_2_ in the context of training load, the present study aims to compare intensity calculations using SmO_2_ and speed in (m/min) during two differentiated physical tasks (Multiple Shuttle Run Test (MSRT) and a 3000-m time trial). The following hypotheses were formulated: (a) Null hypothesis (H0): Training load calculations are similar between differentiated tasks (40 m MST and 3000 m time trial) and (b) Alternative hypothesis (H1): Training load calculations are not similar between differentiated tasks (40 m MST and 3000 m time trial).

## 2. Methods

### 2.1. Experimental Approach to the Problem

This study utilized a randomized crossover design approach. The aim was to compare the training load calculation based on distance, and time as external indicators and HR and SmO_2_ as internal indicators. Subjects visited the athletic track on 2 separate occasions (first familiarization and second evaluation) for about two hours each session. In each session, athletes performed in a randomized order a 10 × 40-m maximal shuttle run test and a 3000-m time trial with 10 min transition between tests. HR and SmO_2_ were obtained to evaluate internal load by portable HR monitors and NIRS portable devices, while total distance, total time, average and decrease speed (fatigue) were obtained to evaluate external load by photocells and tape measure. From the combination of internal (HR, SmO_2_, desaturation rate and cardio-muscular oxygen index, CMOI) and external load variables (distance/time = average speed), training load (intensity) and Eff_index_ were calculated. All calculations were derived from the data obtained during testing.

### 2.2. Subjects

A total of 29 university athletes (male, *n* = 18; female, *n* = 7) participated voluntarily in the present study (age: 21.4 ± 3.2 years; height: 177.1 ± 4.5 cm; weight: 67.8 ± 6.2 kg). Their training experience in running and sprint ranges from 1 to 4 years, as they are university-level athletes. Each athlete initially completed a medical history questionnaire and signed an informed consent form. Participants met the following inclusion criteria: (a) ≥18 years old, and (b) perform a minimum recreational activity (i.e., in the past 6 months at least 3 h of aerobic exercise per week). On the other hand, they were excluded from participation if they reported or exhibited: (a) medical history that could affect results (including cardiovascular, metabolic, pulmonary, renal, hypertension or musculoskeletal impairments), (b) use of medication affecting muscle oxygenation, (c) pregnancy, and (d) daily use of ergogenic aids or sports dietary supplements within 6 weeks before the study. Subjects were asked to maintain normal levels of physical activity/exercise during participation. This study was approved by the Bioethical Review Board of the Institution (Number code: 131/2018). A signed consent was obtained from each subject prior to their participation.

### 2.3. Procedures

The study was composed of two sessions, separated between almost 72 h to avoid fatigue. Trials were administered over one week between 9:00 a.m. and 1:00 p.m. in general 20 °C to 30 °C weather, and after at least 48 h without strenuous physical activity or exercise. In the first session, participants performed a familiarization session with the two tests (10 × 40-m maximum shuttle run test and 3000-m time trial test). During the first session, athletes were familiarized with high monitoring (heart rate bands and NIRS portable devices). In the second session, subjects realized the evaluation session.

Previous to the trials, participants performed the following protocol: (1) athletes arrived 45 min before the assessment to place the near-infrared spectroscopy (NIRS) sensor on the athlete’s dominant leg and prepared the native software, heart rate monitors, and speed photocells at 20 m for MST testing; (2) participants completed a standardized warm-up consisting of 5 min on a stationary ergometer bike, followed by 5 min of supervised dynamic exercises (walking lunges, jogging, heel lifts, high knees, and leg swings); (3) subjects performed two 40-m shuttle runs at 60% and 80% effort with 1 min of recovery between bouts; and (4) before beginning the main exercise protocol, they performed 5 min of seated rest (no data collected during this time).

The trials were randomly administered in 2 groups: (1) one group performed the 40-m MST followed 10 min later by a 3-km run, and (2) the other group completed the 3000-m run first, followed by 10 min of rest by the 40-m MST. On the second session, the trials were reversed to prevent order bias. Outcome performance variables included time to completion, sprint times (MST), time for each 400 m (3000 m), and estimated speed (m/s), while hemodynamic measures included heart rate and muscle oxygen saturation percentage (SmO_2_). The test protocols were administered by the same researchers for all visits. All subjects were instructed to maintain habitual physical activity and dietary intake during the week prior to testing.

### 2.4. Assessment Protocols

Anthropometric Equipment. Standing height was measured for each subject using a wall-mounted stadiometer (SECA, Hamburg, Germany). Body mass was assessed using a digital scale (Model BC-601; TANITA Corporation, Tokyo, Japan).

40-m Maximal Shuttle Run test (MST). Participants began the test from a line 30 cm behind the start line (to avoid false triggering of the first timing gate) with times electronically recorded via Chronojump photocell^®^ (Chronojump, Barcelona, Spain) double-beam photocells and Chronojump software version 1.7.1.8 (Chronojump, Barcelona, Spain) for Mac. For the 40-m MST, subjects ran back and forth between two lines placed 20 m apart, with the start/finish line (and photocells) positioned at the midpoint of the course. Per instructions, each subject ran 10 m from the start/finish line to the far end of the course, turned 180°, ran 20 m to the other end of the course, turned 180°, and ran 10 m back to the start/finish line. Subjects were instructed to place at least one foot on the line at the end of each shuttle, which was monitored for full compliance [22,23]. Recovery between each sprint shuttle was 30 s, totaling 210 s of recovery and 400 m of effort distance. The intraclass correlation coefficient between the familiarization test and the test was 0.93 and a Coefficient of variation of 4.7%.

3000-m Time Trial. Participants completed a maximal 3000 m time-trial on an official 400-m outdoor synthetic athletics track (7.5 laps). The 3000 m distance was chosen as it is run at velocities associated with maximum oxygen uptake (VO_2_max) and has been previously shown to be a reliable indicator of running performance in trained runners [24]. Participants were familiarized with the protocol and were blinded to pace and time during the effort. Race time was recorded via timing chips. Completion of the full 3000 m was mandatory for each athlete, with split times recorded at 200 m, 600 m, 1000 m, 1400 m, 1800 m, 2200 m, 2600 m and 3000 m. The intraclass correlation coefficient between the familiarization test and the test was 0.96 and a Coefficient of variation of 2.4%.

*External load.* External load indicators were derived from distance and time parameters (MST: 400 m; 3 km time trial: 3000 m). To analyze each repetition or split, the time of each segment was recorded. Additionally, the fastest repetition or split was identified (MST: time per repetition; 3 km time trial: time per 7 × 400 m leg, excluding the initial 200 m to avoid biased averages). To determine average speed, calculations were performed by dividing the total time by the number of repetitions (MST: total time/10 repetitions; 3 km time trial: total time/7.5 laps). Finally, fatigue was quantified as the percentage decrease in sprint or split performance during subsequent tests, as per the following formulas:

MST, Fatigue(%)=(100 × (total time ÷ (10×ideal time))−100

3 km time trial, Fatigue(%)=(100 × (total time ÷ (7.5×ideal time))−100

where the “total time” represents the sum of the sprint times or split and the “ideal time” represents the sum of the time based on the fastest sprint or laps.

*Internal load—Heart rate.* The maximum heart rate (HR_MAX_) reached in each test (MST: 10 values, one per repetition; 3-km time trial: 7 values, one per split) as well as heart rate recovery (HRR) were registered. HR was recorded using a chest strap monitor (HRM-Tri, GarminTM, Olathe, KS, USA) paired with a wrist-worn smartwatch (Forerunner 735xt, GarminTM, Olathe, KS, USA) using ANT+ technology, with a sampling frequency of 4 Hz. The heart rate strap was positioned at the level of the xiphoid process and adjusted using a strap system. Values were then converted to % of HR_MAX_, using the maximum reached in either trial as reference:

$$\%\;{HR}_{MAX}=\left({HR}_{MAX}\;each\frac{repetition}{split}÷\;{HR}_{MAX}\;all\frac{repetitions}{splits}\right)\times 100$$

Internal load—Muscle oxygen saturation. SmO_2_ was monitored using a NIRS device with a sampling frequency of 1 Hz (MOXY, Fortiori Design LLC, Minneapolis, MN, USA). The device was securely fastened to the gastrocnemius muscle using an elastic dark strap to prevent light contamination artifacts and movement. The exact location of the portable NIRS monitor was 18–20 cm below the knee, parallel to the major axis of the gastrocnemius. The gastrocnemius muscle also has good reproducibility for measuring SmO_2_, being a muscle with good aerobic capacity in the lower limbs [25,26]. Minimum and maximum SmO_2_ values were the main NIRS study variables as they reflect oxygen consumption and recovery between series, and are suitable for multiple sprint studies [16].

Two variables were calculated by SmO_2_: (1) the percentage of muscle oxygen extraction (∇% SmO_2_) from the SmO_2_ desaturation and re- saturation values was obtained during each effort using the difference between SmO_2_ start (1 s before starting each effort) and SmO_2_ stop (last second of the work interval) [16,27]; and (2) desaturation rate that it was utilized previously by Vasquez-Bonilla et al. [20] and was calculated as the difference between the maximum SmO_2_ value during the work interval and the minimum SmO_2_ value during the rest interval, divided by the duration of the work interval.

$$MST,\;\nabla \%{SmO}_{2}=(((minimum\;{SmO}_{2}\times 100)\;÷\;initial\;{SmO}_{2})-100)\times -1$$

$$3\;km\;time\;trial,\;\nabla \%{SmO}_{2}=\left(\left(minimum\;{SmO}_{2}\times 100)÷\;85\%\;{SmO}_{2}\right)-100\right)\times -1$$

$$\;\;MST,\;Desaturation\;rate\;\left(\%/s\right)=\left({SmO}_{2}\;maximum-{SmO}_{2}\;minimum\right)÷\;sprint\;time\;(s)$$

$$3\;km\;time\;trial,\;Desaturation\;rate\;\left(\%/s\right)=\left(85\%\;{SmO}_{2}-{SmO}_{2}\;minimum\right)÷\;sprint\;time\;(s)$$

For the ∇%SmO_2_ and desaturation rate calculations for the 3000 m, 85% was used as the maximum SmO_2_ value, since 100% SmO_2_ is not a normalized value and in practice a value above 80–82% SmO_2_ can be used, which is where a muscle is considered fatigued at rest [28]. This was done because the ascending slope and resaturation cannot be obtained for the continuous 3000 m exercise. 

*Internal load—Cardio-Muscular Oxygen Index.* It represents the athlete’s hemodynamic response and was captured using heart rate and muscle oxygenation data. This could contribute to integrative training load models seeking to find central and peripheral adaptations or exercise economy outcomes [29]. It also simulated the oxygen pulse formula (VO_2_/HR) [30]. This formula represents the amount of oxygen extraction work at the percentage of heart rate intensity, and is a measure of cardiovascular efficiency in delivering oxygen to tissues per heart beat. This is based on the study of Murias et al. [31] that used the ratio of mean HHb with NIRS. The formula was expressed as follows:

$$Cardio-Muscular\;Oxygen\;Index\left[CMOI\left(\%\right)\right]=\left(\nabla \%{SmO}_{2}÷\%{HR}_{MAX}\right)\times 100$$

Training load. To assess training load, an integrative model of external and internal load indicators was designed [1,7]. Intensity was calculated as the product of external load and internal load (EL × IL) as indicated by Bourdon et al., Campos-Vázquez et al. and Schimpchen et al. [29,32,33]. Likewise, Eff_index_ values were calculated from the relationship between external load and internal load (EL/IL) as shown by Lima-Alves et al., Schimpchen et al., and Staunton et al. [6,29,34]. The interactions were shown in Table 1.

### 2.5. Statistical Analysis

First, descriptive data of mean (m) and standard deviation (SD) were presented along with a Shapiro-Wilk test of normality. After verifying the normality of the data, a paired *t*-test was applied. To compare the likelihood of H0 vs. H1, a Bayesian paired-samples *t*-test with a preset Cauchy prior value of 0.707 (centered at zero) was utilized to ascertain whether the training load estimates were similar between the 40-m (MST) and the 3000-m time trial run. A default prior of 0.707 was chosen based on previous recommendations [35,36]. Bayes factors (BF^10^) were used to provide evidence for (BF^10^ of ≤0.33) or against (BF^10^ of ≥3.0) the null hypothesis. Additionally, the following thresholds for Bayes factors were provided: <0.01, decisive evidence for null hypothesis; 0.03–0.01, very strong evidence for null hypothesis; 0.1–0.03, strong evidence for null hypothesis; 0.3–0.1, moderate evidence for null hypothesis; 1–0.3, anecdotal evidence for null hypothesis; 1, no evidence; 1–3, anecdotal evidence for alternative hypothesis; 3–10, moderate evidence for alternative hypothesis; 10–30, strong evidence for alternative hypothesis; 30–100, very strong evidence for alternative hypothesis; >100, decisive evidence for alternative hypothesis [36]. Cohen’s d effect sizes (ES) [37] where 0.2, 0.5 and 0.8 represent small, medium and large effect sizes respectively. All data was analyzed in JAMOVI 2.2 (The Jamovi Project, 2020).

## 3. Results

Table 2 shows the results of the external load between the 40-MST and 3000 m. Statistical differences were found for time in seconds (percent change, Δ = 1093%, *p* < 0.001), fatigue index (Δ = 2242%, *p* < 0.001), and velocity covered in meters per minute (Δ = 33%, *p* < 0.001). Likewise, a large effect size was observed across all external load indicators.

The Bayesian factor results in Table 3 show differences in internal load between the 40-MST and 3000 m for the variables HR_MAX_ (%) (Δ = 8%, *p* = 0.002), average SmO_2_ (Δ = 50%, *p* < 0.001), and desaturation rate (Δ = 80%, *p* < 0.001). Average SmO_2_ and desaturation rate also had a large effect size (ES), while HR_MAX_ had a medium ES. Likewise, no probability differences were observed for the variables HR (bpm) (Δ = 5%, *p* = 0.150), ∇%SmO_2_ (Δ = 5%, *p*-value = 0.612), and CMOI (Δ = 22%, *p* = 0.039). HR (bpm) and CMOI had a medium ES, while ∇%SmO_2_ had a small effect.

The results of intensity in training load indexes are shown in Table 4. The Bayesian factor found statistical differences between 40-MST and 3000-m in m/min × %HR_MAX_ (Δ = 33% *p* < 0.001), m/min × ∇%SmO_2_ (Δ = 30% *p* = 0.003), m/min × desaturation rate (Δ = 87% *p* < 0.001), m/min × CMOI (Δ = 45 % *p* < 0.001). Likewise, all workloads had a large effect size (ES), except m/min × ∇%SmO_2_ which obtained a medium ES.

The Bayesian factor results in Table 5 show differences in efficiency indices between the 40-MST and 3000-m for the variables m/min ÷ %HR_MAX_ (Δ = 25%, *p* < 0.001), (m/min ÷ ∇%SmO_2_) (Δ = 40%, *p* < 0.001), and (m/min ÷ desaturation rate) (Δ = 225%, *p* < 0.001) with a large effect size (ES). However, for m/min ÷ CMOI (Δ = 22%, *p* = 0.035) no probability differences were observed, with only anecdotal evidence in favor of the alternative hypothesis but with a significant *p*-value and medium ES.

## 4. Discussion

This is the first study showing formulas to calculate training load using SmO_2_ as an internal load parameter along with speed (m/min) as an external load indicator, comparing two different exercise types, the 40-m MST considered high-intensity interval and a 3000-m time trial. The main finding of this study is the methodology to calculate intensity with practical application of calculations based on SmO_2_, which is a variable that can be used to determine skeletal muscle bioenergetics and hemodynamics.

In the context of external load, the MST test is a Repeated-Sprint protocol used to induce neuromuscular fatigue [38]. It was also assumed that running the 3000-m race at a relatively high intensity and hard work should cause significant changes in inflammation markers [39]. While it’s true that one trial can influence the subsequent fatigue of the next task, it’s important to consider that the disparities in external load over time, fatigue, and speed presented in Table 1 should be viewed as the main contrast in the total load of each trial, particularly without considering the transition between trials as indicated in similar studies [40]. Since the MST represents more intermittent work dependent on high speed that uses anaerobic glycolysis and phosphate recovery as fuel, contrary to the 3000-m run which depends on maintaining speed at maximal aerobic capacity zones and the fuel is to a lesser extent fat and carbohydrate oxidation [41]. However, performance in both tests is largely determined by the potential for oxidative energy supply in the active muscles. These statements should be expressed with internal load markers like HR and more objectively SmO_2_ observed in Table 2.

In general, systemic oxygen volume is recognized as a gold standard indicator to prescribe training at different intensities and SmO_2_ could be a peripheral oxygenation indicator. In the final stage of cellular respiration, the mitochondria use oxygen (O_2_) as an energy exchange source, supplied through energy substrates glucose, fatty acids and amino acids that undergo catabolism to feed into the tricarboxylic acid (TCA) cycle, which generates substrates for the electron transport chain (ETC) within the muscle [42]. Additionally, SmO_2_ changes depend to a large extent on the phosphocreatine system and its restoration during exercise (*r* = 0.980) [43], Therefore, it is not absurd to think that desaturation and resaturation changes depend largely on oxidative phosphorylation [44,45]. For oxidative phosphorylation to occur, nicotinamide adenine dinucleotide (NADH) and flavin adenine dinucleotide (FADH_2_) molecules must exist in the electron transport chain that carries oxygen; this is the final and most efficient process of ATP production in cellular respiration.

In this study, we observed that SmO_2_ values during MST are lower than 3000-m values, meaning the 40-m MST test was more intense because more glycolytic work was used and therefore more pronounced muscular oxygen use than in the 3000-m test, but ∇%SmO_2_ values were similar (Table 2). This is due to the methodological difference in the calculations of each variable. To explain these data that can be considered anomalous at first, first consider that SmO_2_ cannot be evaluated with the raw value brought by NIRS devices (0–100% scale) without proper interpretation of hemodynamics about blood flow changes. A clear example is that SmO_2_ has different physiological behavior when the exercise is high intensity intermittent compared to continuous exercise like a time trial [11]. SmO_2_ values need reassessment for interpretation, as shown in this manuscript with the inclusion of SmO_2_ data during effort and recovery (∇%SmO_2_) indicating how much oxygen is used per repetition, similarly desaturation and resaturation rates can be used, showing SmO_2_ in relation to interval duration. The desaturation rate (%/s) was higher in 3000-m than 40-m MST because it depends on the time of each interval; each 3000-m interval was 400 m, while MST was 40 m per sprint, therefore more time was traveled per interval in 3000-m. Another option to assess muscle oxygen in continuous running like 3000-m is to calibrate SmO_2_ values with arterial occlusion to obtain true minimum SmO_2_ [46]. We consider it more practical for coaches to set a limit value like 85% SmO_2_, because values above this limit are not considered any physical effort [47] since values above 80–85% are often a product of acute fatigue hyperemic response [28]. Therefore, these interpretations and data should be introduced into formulas for oxygen loss (∇%SmO_2_) and desaturation during continuous exercise, but ultimately affect comparisons with other exercise types as resulted in this study.

On the other hand, the ∇%SmO_2_/%HR_MAX_ ratio (CMOI) can provide hemodynamic and cardiovascular data in response to exercise. Increased heart rate during high intensity will cause oxygen transport to increase; however, O_2_ supply and demand for muscle contraction will depend on the oxidative capacity of the athlete [48]. HHb measured by NIRS can indicate peripheral oxygen extraction and is related to VO_2_ (*r* = 0.91); therefore it becomes interesting to jointly evaluate these variables from values greater than >85% HR_MAX_, which are at the VT2 limit, where fatigue work begins. What was observed in this study is that variables derived from SmO_2_ differed in the two exercise types [16] and HR did not, as HR influenced CMOI, obtaining results in favor of the null hypothesis (FB^10^ = 1.861). It is evident that the HR_MAX_ reached by each individual is completely personal and influenced by the central nervous system (but environmental conditions can also influence it (temperature, humidity, altitude, etc.) making it difficult to find intensity difference in these exercise types [15,49]. Finally, to consider CMOI, SmO_2_ should be seen as a complement that identifies changes that cannot be detected with HR, for example in uphill and downhill running [15], even so they are parameters that influence each other. From a physiological point, recovery depends not only on cardiac stress but on the individual’s re-oxygenation capacity [50].

**Figure 1:**
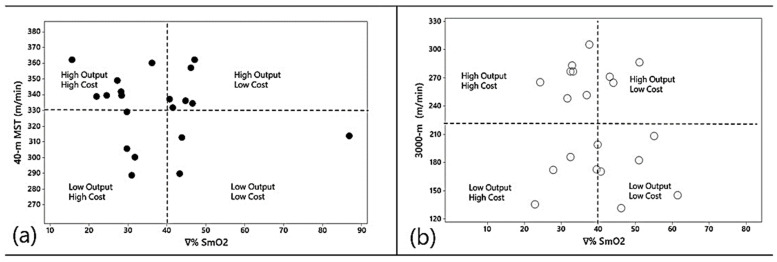
Description of ∇%SmO_2_ for individual monitoring during two different tasks (40-m MST vs. 3000-m trials). The use of this graph represents four dimensions of intensity: (1) High Output-High cost; (2) High Output-Low cost; (3) Low Output-High Cost and (4) Low Output-Low Cost. This method illustrates the intensity in a practical manner using the average values of the external and internal load, as indicated by the authors Ammann et al. [52]. Graph (**a**) shows the individual values from the 40-m MTS task and graph (**b**) shows the individual values from the 3000 m time trial.

In the 40-m MST test, as is logical the training load was higher in all variables because multiplying external load by internal load reflects the product of the work. However, it must be considered with care when interpreting SmO_2_ data. As shown in Figure 1, a lower ∇%SmO_2_ is an indicator of more intense work for both the 40 m MST and 3000 m trials and can interfere with the training load of each athlete [51]. However, the trials cannot be directly compared to each other, since a lowering of ∇%SmO_2_ may reflect effective intense work for the 40-m MST but not as much for the 3000-m run. Rather, the use of ∇%SmO_2_ should be interpreted as a variable that identifies the type of work, which can be more anaerobic or more aerobic metabolism [10]. However, this can only be determined when comparing the same trial in the same individual [1]. Finally, the Eff_index_ variables demonstrated how subjects obtained a different stimulus in the two trials, and Eff_index_ shows the exercise economy of an individual when exposed to an external load [6,29,34]. This is the first demonstration of SmO_2_ data to calculate Eff_index_.

Although the present study provides the first approach in the use of SmO_2_ as an internal load indicator to calculate training load (intensity and Eff_index_), the different limitations should be acknowledged. A major limitation is that currently no devices provide the SmO_2_ data as shown here, which can vary the methodology. Spreadsheets would need development (see Appendix A) to calculate metrics from SmO_2_. Furthermore, no inertial systems were utilized to quantify external load metrics like player load. Incorporating these systems would have strengthened associations between internal and external loads. Finally, the linear running tests differ in metabolic systems but do not involve multidirectional movements seen in field/court sports. Further studies should implement these SmO_2_-based calculations in team sports (e.g., soccer, basketball) with complex movement patterns. Additionally, training load studies utilizing SmO_2_ require a longitudinal design to detect fatigue thresholds and performance improvements over time.

## 5. Practical Applications

Fitness coaches and strength and conditioning trainers can apply the findings from this research to monitor and adjust training intensity and load, utilizing SmO_2_ variables for insight. For SmO_2_ there are different interpretation methods: (1) observing a SmO_2_ and ∇%SmO_2_ decrease may indicate a more anaerobic and intense exercise; but (2) a lower desaturation rate may signify lower training load relative to time and speed. In addition, any variable calculated through SmO_2_ can be utilized since all SmO_2_ parameters are interrelated. Hence, coaches should have criteria based on muscle oxygen physiology in response to the exercise type. A practical application for training load monitoring is shown in Figure 1, which can identify training session volumes and intensity based on the average group response and four intensity dimensions: (1) High Output-High Cost; (2) High Output-Low Cost; (3) Low Output-High Cost and (4) Low Output-Low Cost. Finally, as stated by Perrey and Ferrari [13], SmO_2_ is characterized by faster feedback compared to VO_2_ and is a more precise parameter than HR, which underestimates the internal response in short duration and high-intensity activities. In this sense, HR has a linear response while SmO_2_ detects small changes at high intensities.

## 6. Conclusions

This study presents intensity calculations based on SmO_2_ as an internal load indicator along with speed as an external load indicator during two differentiated exercises (40-m MST and 3000-m running trial). Assessing m/min together with ∇%SmO_2_ could be a good strategy to calculate the intensity and training load of a specific exercise or task. Researchers cannot base their interpretations solely on the SmO_2_ value provided by NIRS, which is meaningless due to the metabolic and vascular factors influencing exercise. Therefore, the type of exercise and the individual subject response must be considered, as these condition the interpretation of SmO_2_.

## Figures and Tables

**Table 1:** Training load indexes based on internal and external load demands.

Intensity (Arbitrary Units)	Eff_index_ (Arbitrary Units)
[(m/min) × HR_MAX_ (%)]	[(m/min) ÷ HR_MAX_ (%)]
[(m/min) × (∇% SmO_2_)]	[(m/min) ÷ (∇% SmO_2_)]
[(m/s) × Desaturation rate (%/seg)]	[(m/s) ÷ Desaturation rate (%/seg)]
[(m/min) × CMOI (%)]	[(m/min) ÷ CMOI (%)]

**Table 2:** Comparison of external load indicators between the maximal shuttle run test and the 3000-m test.

External Load Indicators	Test	Mean	SD	BF_10_	±%	*p*	ES
Total time (s)	40-MST	72.74	5.21	1.98 × 10^−20^	2.88 × 10^−24^	<0.001 *	14.55
	3000 m	871.80	247.80				
Fatigue Index (a.u.)	40-MST	7.74	4.80	1.46 × 10^+6^	9.15 × 10^−13^	<0.001 *	2.17
	3000 m	164.24	71.02				
Average speed (m/min)	40-MST	331	22.8	6.25 × 10^+6^	1.32 × 10^−13^	<0.001 *	2.39
	3000 m	222	56.8				

Note. SD: Standard deviation; ES: Effect size; * *p*-value < 0.05 statistically significant. Bayesian factor (BF^10^) = evidence in favor (BF^10^ of ≤0.33) or against the null hypothesis (BF^10^ of ≥3.0). Qualitative interpretation of the Effect sizes (ES): Small = 0.20–0.49; Median = 0.50–0.79; and large => 0.80.

**Table 3:** Comparison of internal load indicators between the maximal shuttle run test and the 3000-m test.

Internal Load Indicators	Test	Mean	SD	BF_10_	±%	*p*	ES
HR (bmp)	40-MST	164.4	12.7	0.609	6.51 × 10^−5^	0.150	0.335
	3000-m	156.1	25.1				
HR_MAX_ (%)	40-MST	94.9	2.2	18.33	3.23 × 10^−6^	0.002 *	0.790
	3000-m	86.5	11.9				
SmO_2_	40-MST	34.0	11.2	3669	1.39 × 10^−9^	<0.001 *	1.379
	3000-m	51.7	8.6				
∇% SmO_2_	40-MST	37.189	14.881	0.262	2.14 × 10^−4^	0.612	0.115
	3000-m	39.155	10.184				
Desaturation rate (%)	40-MST	2.807	1.236	167,290	4.94 × 10^−13^	<0.001 *	1.867
	3000-m	0.558	0.109				
CMOI (%)	40-MST	39.20	15.44	1.70	2.99 × 10^−5^	0.039 *	0.496
	3000-m	30.51	8.67				

Note. HR: heart rate; HR_MAX_: maximum heart rate; SmO_2_: muscle oxygen saturation ∇% SmO_2_: percentage of muscle oxygen extraction; CMOI: cardio-muscular oxygen index; SD: Standard deviation; ES: Effect size; * *p*-value < 0.05 statistically significant. Bayesian factor (BF^10^) = evidence in favor (BF^10^ of ≤0.33) or against the null hypothesis (BF^10^ of ≥3.0). Qualitative interpretation of the Effect sizes (ES): Small = 0.20–0.49; Median = 0.50–0.79; and large => 0.80.

**Table 4:** Comparison of intensity between the maximal Shuttle run test and the 3000-m test.

Workload (Arbitrary Units)	Test	Mean	SD	BF_10_	±%	*p*	ES
(m/min) × HR_MAX_ (%)	40-MST	314.65	24.281	5.08 × 10^+6^	2.68 × 10^−13^	<0.001 *	2.332
	3000-m	210.00	53.435				
(m/min) × (∇% SmO_2_)	40-MST	122.65	47.61	12.5	4.36 × 10^−6^	0.003 *	0.746
	3000-m	85.77	27.40				
(m/s) × Desaturation rate (%/s)	40-MST	15.55	6.77	583,387	1.32 × 10^−12^	<0.001 *	2.040
	3000-m	2.06	0.85				
(m/min) × CMOI (%)	40-MST	129.27	49.446	169.6	1.17 × 10^−8^	<0.001 *	1.040
	3000-m	70.63	32.98				

Note. HR_MAX_: maximum heart rate; ∇% SmO_2_: percentage of muscle oxygen extraction; CMOI: cardio-muscular oxygen index; SD: Standard deviation; ES: Effect size; * *p*-value < 0.05 statistically significant. Bayesian factor (BF^10^) = evidence in favor (BF_10_ of ≤0.33) or against the null hypothesis (BF^10^ of ≥3.0). Qualitative interpretation of the Effect sizes (ES): Small = 0.20–0.49; Median = 0.50–0.79; and large => 0.80.

**Table 5:** Comparison of efficiency index between the maximal Shuttle run test and the 3000-m test.

Workload (Arbitrary Units)	Test	Mean	SD	BF_10_	±%	*p*	ES
(m/min) ÷ HR_MAX_ (%)	40-MST	3.49	0.235	5197.999	8.84 × 10^−10^	<0.001 *	1.419
	3000-m	2.60	0.717				
(m/min) ÷ (∇% SmO_2_)	40-MST	10.19	4.17	137.03	1.07 × 10^−8^	<0.001 *	1.016
	3000-m	6.06	2.21				
(m/s) ÷ Desaturation rate (%/seg)	40-MST	2.25	0.815	1.80 × 10^+10^	2.91 × 10^−17^	<0.001 *	3.912
	3000-m	7.31	0.994				
(m/min) ÷ CMOI (%)	40-MST	9.69	4.115	1.861	3.17 × 10^−5^	0.035 *	0.509
	3000-m	7.55	1.87				

Note. HR_MAX_: maximum heart rate; ∇% SmO_2_: percentage of muscle oxygen extraction; CMOI: cardio-muscular oxygen index; SD: Standard deviation; ES: Effect size; * *p*-value < 0.05 statistically significant. Bayesian factor (BF^10^) = evidence in favor (BF^10^ of ≤0.33) or against the null hypothesis (BF_10_ of ≥3.0). Qualitative interpretation of the Effect sizes (ES): Small = 0.20–0.49; Median = 0.50–0.79; and large => 0.80.

## Data Availability

The data presented in this study are available on request from the corresponding author.

## Appendix A. Procedure for Calculating Training Load by Muscle Oxygen Saturation

The provided text illustrates a procedure for calculating training load and intensity using a near-infrared spectroscopy (NIRS) sensor that measures muscle oxygen saturation.

Training Load Calculation:

To compute the training load, several sequential steps need to be followed. Firstly, understanding the Muscle Oxygen Saturation scale, which spans from 1% to 100%, indicating a range between 45–75% of SmO_2_ or normal resting values. Changes in SmO_2_ levels reflect exercise intensity, while recovery or resultant hyperemia is indicative of active recovery time during a specific task or exercise.

**Step 1**

Calculation of External Load:

Consider an athlete undertaking a 40-m Maximal Shuttle Run Test (MST, 10 × 40-m with 30 s recovery between sprints) and yielding the following data:

**Table A1:** Individual speed values during sprint-repeated (40-MST).

Nº	Sprint_1	Sprint_2	Sprint_3	Sprint_4	Sprint_5	Sprint_6	Sprint_7	Sprint_8	Sprint_9	Sprint_10
Time (s)	6.62	6.33	6.54	6.74	6.74	7.01	6.72	6.66	6.42	6.50
Speed (m/s)	6.04	6.32	6.12	5.93	5.93	5.71	5.95	6.01	6.23	6.15

Total time score: 66.28 s for 400 m

Average speed: 6.04 m/s for 400 m

The external load is computed using the formula [(distance(m) ÷ time(min)]:

External Load = [400 ÷ (66.28 ÷ 60)]

External Load = 362 (m/min)

**Step 2**

Calculation of Internal Load:

Utilizing a NIRS device, the athlete measures SmO_2_ in the gastrocnemius muscle during sprints and recovery periods (30 s). Sampled values for sprints and the recovery are recorded:

**Table A2:** Individual SmO_2_ values during sprint-repeated (40-MST).

	Sprint_1	Sprint_2	Sprint_3	Sprint_4	Sprint_5	Sprint_6	Sprint_7	Sprint_8	Sprint_9	Sprint_10
SmO_2_ of Sprint work	18	21	18	17	11	21	25	27	28	28
SmO_2_ of Recovery	38	42	40	30	26	35	41	34	52	39

SmO_2_ of Sprint work: Ranging from 11 to 28%

SmO_2_ of Recovery: Ranging from 26 to 52%

Average values obtained: SmO_2_ of sprint work = 21.4% and SmO_2_ of recovery = 37.7%

Internal Load Calculation using ∇%SmO_2_:

Oxygen loss (∇%SmO_2_) = ((SmO_2_ of Sprint work × 100) ÷ (SmO_2_ of recovery − 100) × −1)

∇%SmO_2_ = (21.4 × 100) ÷ (37.7 − 100) × −1

∇%SmO_2_ = 34.3%

Additionally, the % of heart rate maximum (HRmax %) during the 40-m Maximal Shuttle Run Test is considered:

**Table A3:** Individual heart rate values during sprint-repeated (40-MST).

	Sprint_1	Sprint_2	Sprint_3	Sprint_4	Sprint_5	Sprint_6	Sprint_7	Sprint_8	Sprint_9	Sprint_10
HR bmp	145	158	159	162	164	164	166	166	166	166
HR**_MAX_** (%)	87	95	96	98	99	99	100	100	100	100

Average heart rate: 162 bpm and %HR_MAX_: 97.5%

Now we can calculate the cardio-muscle oxygen index (CMOI %):

CMOI %= (∇%SmO2 ÷ %HR_MAX_) × 100

CMOI %= (34.3 ÷ 97.5%) × 100

CMOI % = 35%

**Step 3**

Calculate Training Load:

The training load is the multiplication of the external load by the internal load:

Training Load = [(m/min) × (CMOI)]

The External and Internal load can be divided by 10 to obtain values that are easier to manage and compare between them:

Training Load = (362 ÷ 10) × (35 ÷ 10)

Training Load= 126.7 A.U.

Final response = the training load is 126.7 arbitrary units for the athlete during repeated sprints (40-m MSRT)

**Step 4**

Calculate Exercise Intensity using Efficiency Index:

The efficiency index is the division of the external load by the internal load, and is expressed by the following formula:

Efficiency index = [(m/min) ÷ (CMOI)]

Efficiency index = 362 ÷ 35

Efficiency index = 10.3 A.U.

Final response= The efficiency index is 10.3 arbitrary units for the athlete during repeated sprints (40-m MSRT).

Option B: Desaturation Rate Measure:

The desaturation rate, expressed as (%/s), provides a time-based view of Sprint, calculated using the formula:

Desaturation rate (%/s) = (SmO_2_ of recovery − SmO_2_ of sprint work) ÷ sprint time (s)

Desaturation rate (%/s) = (37.7 − 21.4) ÷ 66.28 s

Desaturation rate (%/s) = 0.24 (%/s)

However, it’s important to note that the desaturation rate solely focuses on time-associated SmO_2_ variations and does not incorporate the external load exposure. Thus, it is considered a time-associated SmO_2_ variable.

This comprehensive process enables a detailed analysis of training load and exercise intensity using NIRS technology, heart rate, and sprint-specific data, providing insights for effective training assessment.
